# Predator-Induced Fleeing Behaviors in Phytoplankton: A New Mechanism for Harmful Algal Bloom Formation?

**DOI:** 10.1371/journal.pone.0046438

**Published:** 2012-09-28

**Authors:** Elizabeth L. Harvey, Susanne Menden-Deuer

**Affiliations:** Graduate School of Oceanography, University of Rhode Island, Narragansett, Rhode Island, United States of America; University of Hull, United Kingdom

## Abstract

In the plankton, heterotrophic microbes encounter and ingest phytoplankton prey, which effectively removes >50% of daily phytoplankton production in the ocean and influences global primary production and biochemical cycling rates. Factors such as size, shape, nutritional value, and presence of chemical deterrents are known to affect predation pressure. Effects of movement behaviors of either predator or prey on predation pressure, and particularly fleeing behaviors in phytoplankton are thus far unknown. Here, we quantified individual 3D movements, population distributions, and survival rates of the toxic phytoplankton species, *Heterosigma akashiwo* in response to a ciliate predator and predator-derived cues. We observed predator-induced defense behaviors previously unknown for phytoplankton. Modulation of individual phytoplankton movements during and after predator exposure resulted in an effective separation of predator and prey species. The strongest avoidance behaviors were observed when *H. akashiwo* co-occurred with an actively grazing predator. Predator-induced changes in phytoplankton movements resulted in a reduction in encounter rate and a 3-fold increase in net algal population growth rate. A spatially explicit population model predicted rapid phytoplankton bloom formation only when fleeing behaviors were incorporated. These model predictions reflected field observations of rapid *H. akashiwo* harmful algal bloom (HAB) formation in the coastal ocean. Our results document a novel behavior in phytoplankton that can significantly reduce predation pressure and suggests a new mechanism for HAB formation. Phytoplankton behaviors that minimize predatory losses, maximize resource acquisition, and alter community composition and distribution patterns could have major implications for our understanding and predictive capacity of marine primary production and biochemical cycling rates.

## Introduction

Blooms of toxic phytoplankton can negatively impact coastal ecosystems and economies through poisoning events that induce high mortality rates in fish [1], birds [2], [3], and illness in humans [4]. Harmful Algal Bloom (HAB) events appear to be increasing in frequency, and it is hypothesized that climate change related alterations of the environment will exacerbate the toxicity of HABs [5]–[7]. Therefore, understanding why phytoplankton in general and many HAB species particularly are successful in forming frequent, large-scale, often mono-specific blooms is of ecological, societal, and economic concern [6], [8].

The balance between growth- and loss-promoting factors largely determines the abundance and distribution of phytoplankton in the marine environment. Research particularly in HAB species has principally focused on mechanisms that impact algal growth rates. Hypothesized causes of HAB formation include vertical migration [9], allelopathy [10], eutrophication [6], and pelagic-benthic life cycles [11]. Increased nutrient availability from land, including agricultural run-off has been considered a chief cause of HABs, but both field and laboratory investigations show diverse HAB nutrient physiology and provide contradictory results between and even within species [12], [13]. Less emphasis has been placed on phytoplankton mortality, specifically the degree with which reduced predation pressure might enable HAB formation [14]–[16]. Predation by unicellular zooplankton (i.e. heterotrophic protists) is the single largest mortality factor for phytoplankton in the ocean, with on average over 50% of daily primary production consumed [17]. Deterrence of heterotrophic protist predators and reduction in predation pressure could therefore significantly enhance phytoplankton survival and thus the potential for bloom formation.

The palatability of a phytoplankton species to a predator depends on factors such as cell size, structure, shape, chemical composition, and nutritional quality e.g. [15]. Consequently, some phytoplankton species are subject to decreased grazing pressure based on intrinsic characteristics of their morphology or physiology. Known predator-induced algal defense mechanisms include changes in morphology [18]–[21] and production of chemical deterrents [22]–[25]. Several studies have reported predator-induced changes in movement behaviors involving metazoan plankton [26], [27], and some dinoflagellates conducted escape jumps to avoid copepod predators [28]. Such predator-induced changes in movements may lead to changes in the population distributions of prey. For instance, the vertical distribution of the freshwater zooplankter, *Daphnia* sp. shifted away from predators or predator exudates [26]. Similarly, the marine copepod, *Acartia hudsonica* changed its vertical distribution and migratory behavior depending on the presence or absence of a common fish predator [29]. The capacity of phytoplankton to exhibit similar, predator-induced avoidance behaviors that result in a shift in population distributions and predation pressure has, to our knowledge, not been examined. Such avoidance behaviors would have ramifications for the abundance and distribution of phytoplankton populations, and thus for the magnitude of matter and energy flow in marine planktonic food webs.

To examine the role of behavior in planktonic predator-prey interactions, we quantified the microscopic cell-cell interactions, population dispersal, and growth rates of *Heterosigma akashiwo* (Raphidophycae, Hada) a common HAB alga in the presence of either the heterotrophic protist predator *Favella sp.* or a range of predator-derived cues in vertically structured 1 L tanks imaged with stereo video cameras that captured microscopic 3D movement behaviors and cm-scale population redistributions at multiple horizons every hour for 12 hours (Figure S1). We found that the presence of an actively grazing heterotrophic protist predator induced avoidance behaviors in phytoplankton movements, which led to an effective population-level avoidance of the predator, and a 3-fold increase in net algal growth rates. Predator-induced phytoplankton fleeing behaviors were essential in model predictions reproducing empirical observations of *H. akashiwo* bloom formation in the coastal ocean.

## Results and Discussion

### Phytoplankton avoidance behaviors

**Figure 1:**
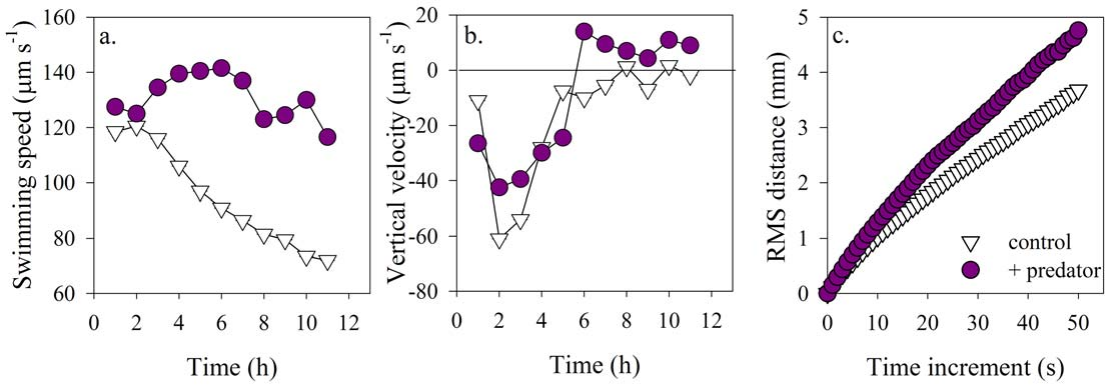
Below halocline movement behaviors. Mean (a) swimming speed (µm s^−1^), (b) mean vertical velocity (µm s^−1^), and (c) root mean square distance (RMSD) (mm) of *H. akashiwo* in the absence (white triangles) and presence of the ciliate predator (purple circles) below the halocline. Error bars here and in all figures are one standard error (SE) of the mean. Frequently SE was small and contained within the symbols. Movement behaviors reflect significantly greater speed and upward motility below the halocline in response to the presence of the predator.

Significant modulation of phytoplankton swimming speed and vertical velocity was observed when *H. akashiwo* was exposed to the actively grazing predator, *Favella* sp. (p<0.001; Fig. 1a & b). Previously reported swimming speeds for this species are strain dependent and range from 50–120 µm s^−1^ [30]. In our experiments, average swimming speed in both the predator exposure and filtered seawater control were high during an initial acclimation period of 2–4 hours. Over the remaining observation period, swimming speed in the control decreased to the strains inherent swimming speed of 70–90 µm s^−1^. However, in the presence of the predator, swimming speed increased by on average 38% and upward vertical velocity was 29% faster compared to the average speed under control conditions. These differences between predator exposure and filtered seawater control were maintained for the entire observation period although the magnitudes changed over time.

**Figure 2:**
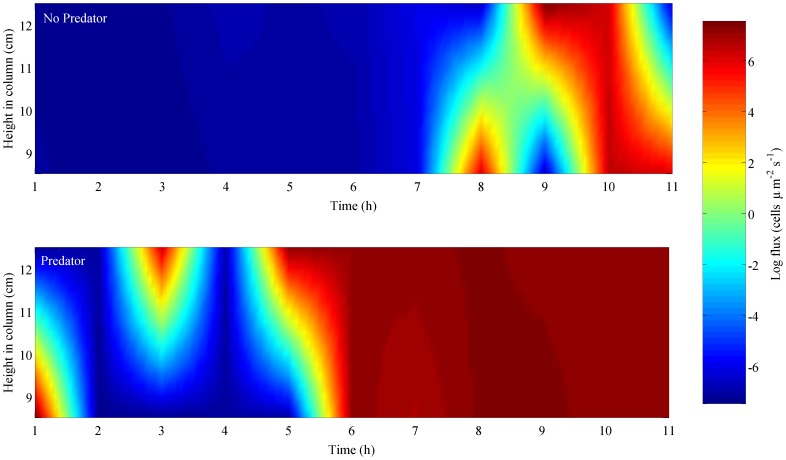
Below halocline vertical flux. Vertical flux (log cells µm^−2^ sec^−1^) of *H. akashiwo*, below the halocline, in the absence (top) and presence (bottom) of the predator. Warmer colors = upward flux and cooler colors = downward flux of phytoplankton cells. After 6 h, in the presence of the predator, the vertical flux of *H. akashiwo* was strongly and persistently upward. In contrast, the vertical flux of *H. akashiwo* in the absence of a predator was initially downward and then directionally inconsistent resulting in no effective change in population distribution.

The presence of a predator resulted in effective modulations of the root mean square distance (RMSD) covered by *H. akashiwo*. RMSD is a useful metric to characterize the diffusivity associated with the cumulative effects of changes in multiple movement behaviors (e.g. swimming speed and turning rate). RMSD helps to distinguish high dispersal rates (i.e. ballistic motion) indicated by RMSD that grows rapidly with time from low dispersal rates (i.e. diffusive motion) indicated by RMSD values that increase at a lower rate [31], [32]. *Heterosigma akashiwo* effectively modulated movements to decrease encounter rates with the predator or predator-derived cues. The most dispersive swimming was observed in response to the presence of the predator (Fig. 1c). Predator-induced changes in individual movement behaviors resulted in a rapid and sustained upward flux of the *H. akashiwo* population (Fig. 2). Vertical migration behaviors are well known for phytoplankton [33], [34], but can be excluded as a causative mechanism here, as expected changes in distribution due to vertical migration would be identical in all treatments. In the predator-free control, phytoplankton population flux was only briefly shifted upwards after 8 h, whereas upward flux commenced immediately in the presence of the predators and after 6 h the entire population was moving upwards.

**Figure 3:**
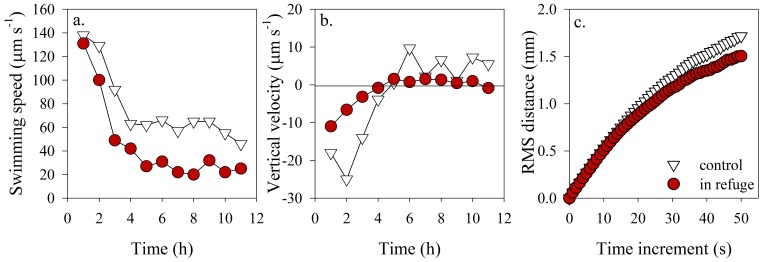
Above halocline movement behaviors. Mean (a) swimming speed (µm s^−1^), (b) vertical velocity (µm s^−1^) and (c) root mean square distance (RMSD) (mm) of *H. akashiwo* in the absence (white triangles) and presence of the predator (red circles) above the halocline. Movement behaviors reflect significantly more retentive swimming above the halocline in response to the presence of the predator below the halocline.

Increases in phytoplankton diffusivity, as indicated by the RMSD, were only observed when algae were directly exposed to the predator, below the halocline. Above the halocline, in a low salinity surface layer (termed refuge) inaccessible to the stenohaline predator, phytoplankton fleeing behaviors vanished. Instead, swimming speed and vertical velocity decreased (Fig. 3 a & b). These differences were maintained over the entire observation period. The increase in speed observed in response to the predator below the halocline was not observed in the refuge. Once cells reached the refuge after predator exposure, the magnitude of the speed underwent a complete reversal and cells swam slower than their inherent swimming speed. As a result, effective dispersal was more retentive within the refuge than in the control (Fig. 3c), resulting in an accumulation of *H. akashiwo* above the halocline.

The ability of *H. akashiwo* to migrate to lower salinity water has been documented in several laboratory experiments [30], [35], [36]. However, this behavior has not been identified to provide a relief from predation. Decreasing co-occurrence with a predator would have a two-fold beneficial effect on *H. akashiwo* survival rates: First, movements away from the predator decrease predator-prey encounter rates and ultimately predation pressure. Second, unlike toxicity that eliminates predators, avoidance behaviors do not remove predators from the system. Therefore, predation pressure is shifted and thus increased on other phytoplankton species that do not display similar anti-predator defenses. This redirected predation pressure would remove competitors from the water column and decrease nutrient competition with other phytoplankton, further increasing the population growth rates of algae displaying these defense behaviors [37]. The capacity to successfully avoid predators may distinguish this species from phytoplankton that are less successful at bloom formation.

### Mechanism of avoidance response

**Figure 4:**
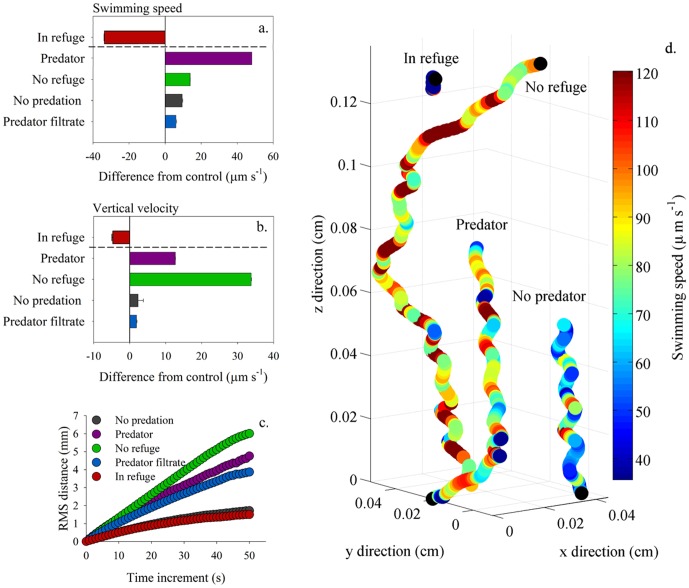
Impact of predator-derived stimuli on movement behaviors. Modulation of phytoplankton movements as a function of predator-derived cues. Difference in (a) swimming speed (µm s^−1^) and (b) vertical velocity (µm s^−1^), above and below the halocline (dotted line), (c) root mean square distance (mm), a proxy of population dispersal rates and (d) characteristic swimming tracks and speeds in the different predator exposure treatments; black circles denote the beginning of a swimming track. Fleeing behavior by the phytoplankton was significant but quantitatively different in response to specific predator-derived cues as evident in swimming metrics, tracks, and dispersal.

We conducted several control experiments with different predator-derived cues to probe for possible mechanisms that explain the induction of phytoplankton fleeing behaviors (Figure S1). First, we exposed *H. akashiwo*, under identical conditions and water column structure to predators that did not feed on this alga [38], [39] thus stimulating *H. akashiwo* with the chemical and mechanical cues of swimming predators, but lacking predation pressure. Second, we exposed *H. akashiwo* to a cell-free filtrate from actively grazing predators, thus removing all mechanical cues. Both these treatments elicited qualitatively identical and significant changes in phytoplankton movements and population fluxes but at a quantitatively lower magnitude than responses observed in the presence of actively grazing predators (p<0.001; Fig. 4). Third, we examined the role of water column structure by removing the low salinity refuge at the top of the tank, forcing a continuous exposure of predator to prey throughout the water column. The presence of the predator in the absence of a refuge elicited a significant increase in swimming speed and vertical velocity in the HAB alga, identical to the fleeing behavior observed in the structured water column; in the absence of a low salinity refuge the difference in vertical velocity from the control was 25% greater in magnitude (p<0.001; Fig. 4). It is noteworthy that under these conditions, the enhanced vertical velocity was in the downward direction, reflecting avoidance of the predator that was aggregated at the top of the tank. Therefore, *Hetetrosigma akashiwo* is capable of fleeing away from the predator, rather than expressing an inherent reaction of moving to the top of the water column in response to predator presence. Treatment specific characteristic differences in individual motility patterns are shown in Figure 4d. Together, these experiments strongly suggest that phytoplankton fleeing behaviors were induced by the presence of predator-derived cues, partially driven by dissolved cues, and quantitatively most significantly driven by the active feeding process.

Both chemical and mechanical stimuli have been reported effective in eliciting phytoplankton responses [22], [28] and exchanges of chemical information within and among trophic levels can directly impact population structure [26], [29], [40]. Interestingly, we observed that *H. akashiwo* exhibited predator-induced avoidance behaviors for an extended period of time, even when *Favella* sp. abundance was low or distant or occurred several hours prior. Neither a mechanical or chemical cue could be responsible for the sustained modulation of phytoplankton movements within the low salinity refuge because the signal could not be effectively transmitted over several cm and hours [41], thus we do not know how this sustained response was elicited. Although we cannot determine the nature of this sustained cue, our observations suggest that either *H. akashiwo* retains information on prior predator exposure for several hours or that cues from conspecifics may have mediated sustained modification of phytoplankton swimming behavior.

### Impact of avoidance on population growth

**Figure 5:**
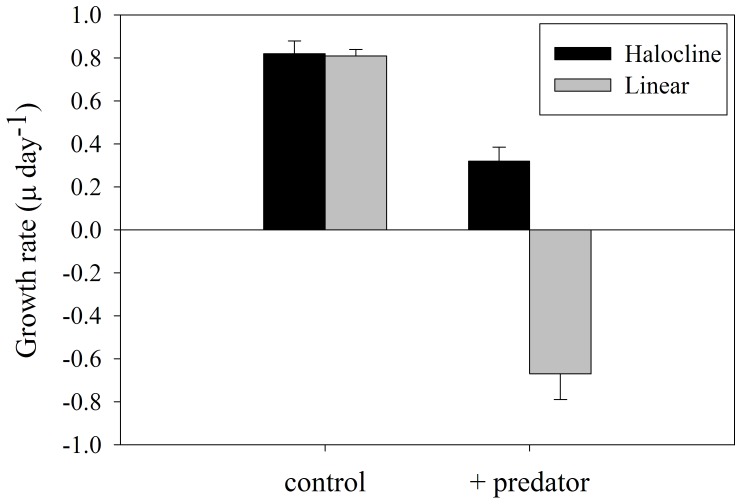
Salinity structure impact on phytoplankton growth rate. Population growth rate (µ day^−1^) of *H. akashiwo* either in a halocline (black) or linear (gray) salinity gradient in the phytoplankton only control (left) and in the presence of the predator (right). Phytoplankton fleeing behaviors and availability of a low salinity refuge effectively reduced predator-prey encounter rates and resulted in significant growth of *H. akashiwo* in the presence of the predator.

To determine the effectiveness of the fleeing behaviors on net population survival rates, we measured phytoplankton population growth rates in the different experimental treatments. With the exception of the presence of the feeding predator, none of the other treatments affected phytoplankton abundances. Under control conditions the HAB alga grew at a rate of μ = 0.81±0.11 day^−1^ (Fig. 5; mean ± SEM), equivalent to a population doubling every 21 hours. In the absence of a low salinity refuge, a forced exposure of predator and prey, the ciliate effectively preyed upon *H. akashiwo* cells, resulting in removal of all of the primary production as well as some of the standing stock, yielding a net negative population growth rate (μ = −0.67±0.12 day^−1^). At this predation rate the entire phytoplankton population would be removed within 28 hours. Fleeing HAB algae, that could access the low salinity refuge, avoided predation resulting in a significantly higher net population growth rate of μ = 0.33±0.06 day^−1^, or a population doubling within 48 hours (p = 0.001). Thus, by invoking the observed fleeing behaviors *H. akashiwo* avoided population elimination as rapidly as within 1 day and attained a considerable population growth rate in the presence of a predator.

The significant increase in fitness imparted by the phytoplankton fleeing behavior suggests that there is little cost associated with this defense. Generally, an anti-predator response is associated with a cost, and organisms are subject to a tradeoff between the benefits and costs of exhibiting specific behaviors [42]. It is well known that *H. akashiwo* is tremendously halo-tolerant and that some strains can maintain maximal growth rates between 5 and 30 psu [13], [43], [44]. Yet, the use of low salinity water as a refuge from predation affords the alga with a competitive advantage because significant positive population growth rates can be maintained in the low salinity surface water due to the halo-tolerance of the alga.

The experimental conditions mimic frequently observed salinity structures in an estuarine setting, including the Fraser River Estuary, British Columbia, Canada [45], [46], where *H. akashiwo* blooms have been documented frequently [47]. Occurrence of salinity structures that include strong haloclines in the field suggests the defense mechanism we observed in the laboratory may be effective in the coastal ocean. In estuarine systems, grazing rate is positively correlated with salinity, increasing from nearshore to offshore [48]. This gradient is established because of the differential salinity tolerance in populations of heterotrophic protists [49], [50]. Moreover, the predation process itself can be negatively affected by low salinities [51]. As a result, *H. akashiwo* is likely exposed to similar concentration gradients in predator abundance as in our halocline experiments. Given *H. akashiwo*'s broad halo-tolerance, there may be little reduction in growth rate in *H. akashiwo* occupying low salinity waters yet the toxic alga would benefit from reduced predator exposure. Thus, the structuring function of salinity combined with these novel predator induced avoidance behaviors provide a tremendous advantage to *H. akashiwo* and would result in significant increases in fitness to this and by implication other phytoplankton that express predator-induced avoidance behaviors.

### Model predictions of HAB formation

**Figure 6:**
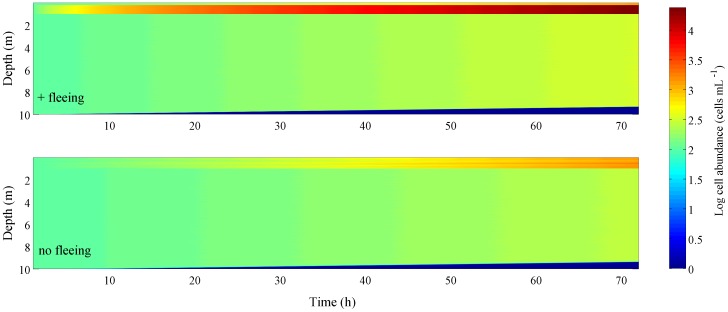
Effect of predator-induced fleeing behaviors on phytoplankton bloom formation. Predictions of phytoplankton abundance and distribution in a spatially explicit model that does (top) and does not (bottom) include empirically observed phytoplankton avoidance of predator inhabited, deeper waters. All parameters, including growth rates were empirically measured and, with the exception of fleeing behaviors, identical in all model runs.

To quantify the ramifications of the observed individual movement behaviors for ecosystem level processes and the formation of HABs, we formulated a spatially explicit, individual based model that predicted *H. akashiwo* abundance in a 10 m water column with a typical salinity profile for areas with near annual outbreaks of *H. akashiwo* HABs [45], [46]. A below-detection limit seed population of *H. akashiwo* (100 cells ml^−1^) that expressed the observed fleeing behaviors attained toxic bloom concentrations of 10^4^ cells ml^−1^ in approximately 2 days; identical simulations that omitted fleeing behaviors predicted bloom concentrations only after 6 days (Fig. 6). Ephemeral occurrences of HAB blooms, including *H. akashiwo* are well known [37], [52] and contribute to their enigma. Although laboratory experiments suggest that *H. akashiwo* can form dense patches at depth through horizontal shear [53], available field data show that *H. akashiwo* blooms occur as surface slicks, where light intensity is highest and salinity lowest [52], [54], [55]. Our data imply that these surface aggregations could form, at least in part, by *H. akashiwo* movements to avoid predators.

### Conclusions

Plankton population dynamics have been difficult to predict and in many cases the species composition has been enigmatic. For example, eutrophication has long been thought to be the driving factor in the formation and persistence of harmful algal blooms e.g. [5]. However, by linking individual movement behaviors with population distributions and dynamics, we show that the consequences of predator-prey interactions in driving localized increases in phytoplankton population abundance may also be important in promoting HAB formation and persistence. Predictions of HAB ecology and management efforts may underestimate population dynamics if they fail to incorporate predator-prey interactions. Moreover, phytoplankton fleeing behaviors may be present in non-HAB phytoplankton and may impact planktonic predator prey interactions in general. These observations highlight the importance of considering phytoplankton motility and modulation thereof, as well as non-lethal (predator avoidance) predator-prey interactions in influencing plankton spatial distributions, population dynamics, and ultimately carbon cycling in the ocean.

## Methods

### Culturing

All experiments were performed on the same strain of the common harmful algal bloom species, the raphidophyte *Heterosigma akashiwo* (CCMP 2809). All phytoplankton cultures were grown in 0.2 µm sterile-filtered autoclaved seawater (FSW), enriched with f/2 nutrients; ciliate predators were grown in FSW only. All cultures were maintained on a 12∶12 h light∶dark cycle, at 15°C, salinity of 30 psu, and a light intensity of 70–80 µmol photon m^−2^ s^−1^ for the phytoplankton cultures and 8–15 µmol photon m^−2^ s^−1^ for *Favella* sp. The cultures were not axenic.

### Experimental Design

To quantify population distributions, movement behaviors, and grazing rate a 30 cm tall, 5.5 cm wide, 1 L octagonal, acrylic observational tank was used. The tank was filled with 1 L FSW using a peristaltic pump. This method allowed for the creation of defined and stable salinity structures and eliminated fluid convection in the chamber [56]. To create a low salinity refuge, the water column was spatially structured to mimic frequently observed estuarine stratification through a halocline with salinities ranging from 8 to 30 psu [36]. To force predator-prey encounter throughout the water column, a linear gradient from 27–30 psu was created in the tank. Source water (300 L) from Narragansett Bay, Rhode Island, USA was collected prior to the experiments at a salinity of 30 psu. The same source water was used in all experiments and for cultures.

Four treatments were used to quantify the effects of predator presence on *H. akashiwo* population distribution and movement behaviors (Figure S1): 1) an algal only control, containing *H. akashiwo* alone, 2) a grazing-predator treatment, containing *H. akashiwo* and a strain of *Favella* sp. that actively grazed on the alga, 3) a predator-exposure treatment of the alga to a non-grazing strain of *Favella* sp., and 4) a test of the response of *H. akashiwo* to chemical cues derived from the actively grazing *Favella* sp. strain. Chemical cues for this treatment were harvested by carefully filtering life predator cultures that were actively feeding on *H. akashiwo* through a 20 µm mesh net. Each treatment was filmed in triplicate. Three separate tanks were used to simultaneously run 1 replicate each of 3 of the 4 treatments and their respective controls. Experiments were repeated until 3 independent replicates of each treatment were acquired. Experiments were initiated at the same time of day to account for diel changes in behavior and physiology. This procedure was repeated for each salinity structure, using predator and prey cultures that were in identical growth conditions and stages.

Using a syringe, organisms were introduced at the bottom of the tank through silicone tubing with an internal diameter of 1 mm. Introduction occurred slowly at a rate of 10 mL min^−1^ to reduce stress to cells and disturbance to the water column. Phytoplankton cells were introduced to the tank first, and allowed to acclimate for 10 min. *Heterosigma akashiwo* was added to the tank for an average final concentration of 180 cells mL^−1^. This concentration was chosen to maximize the duration individual cells could be tracked continuously before overlapping with tracks from other cells. Predator cells were then added to the tank using the same silicone tubing as used for phytoplankton. To minimize the transfer volume, 2 L of ciliate culture (final concentration 50 cells mL^−1^) were gently condensed to 30 mL using a submerged, 20 µm Nitex mesh 15 min before introduction to the bottom of the experimental chamber. For experiments testing the effect of dissolved cues, the *Favella* sp. culture was condensed immediately prior to filling the tank, and gently filtered through a 0.2 µm syringe-tip filter. Due to the ciliates limited halo-tolerance *Favella* sp. distributions were restricted to salinities >15 psu, that is depth below the halocline [39]. To mimic this restricted predator distribution in the dissolved cue treatment *Favella* sp. filtrate was only added to the lower half of the tank. An equivalent volume of water was added to the control tanks, so that volumetrically the treatments remained the same.

To determine *H. akashiwo* growth and predator grazing rates the 1L volume of the tanks were gently homogenized after 24 h incubations and one 30 mL subsample was withdrawn from each tank and preserved with 1% acid Lugol's solution. Ten milliliters of the preserved sample were settled and enumerated using the Utermöhl method [57].

### Video capture and analysis

The methods of video capture and analysis followed those presented in Harvey and Menden-Deuer [39]. Briefly, three-dimensional movement trajectories were derived from stereo-video images, captured at 30 fps for 1 minute at 6–8 horizons equally spaced along the tank, for a total of 12 hours. Image analysis was automated and identical track assembly and analysis parameters were applied to all videos. Predator and prey were distinguished based on their size difference, which was calibrated with the control videos. Only time points that had ≥50 individuals in each replicate, and cells tracked for a minimum of 3 s or longer were used in the movement analysis. All time points were used in abundance analysis.

### Model Specifications

A spatially explicit, individual-based model of *H. akashiwo* abundance and population distribution over time was constructed. The water column was structured by a halocline in accordance with a typical salinity profile for areas where *H. akashiwo* blooms are common [45], [46]. The duration of the simulation was 7 days, evaluated at hourly intervals. The water column was 10 m deep and evaluated at cm increments. Cell growth and mortality occurred at the empirically measured rates. Net population growth rate of *H. akashiwo* was 0.81 d^−1^ above the halocline, and 0.30 d^−1^ in the remainder of the water column, reflecting removal by the predator. Cell motility also followed the empirically measured swimming speeds, turning rates, and vertical velocity. All settings in the two model evaluations were identical, with the exception that one model iteration incorporated the empirically observed fleeing behaviors, whereas the other model incorporated the movement behaviors measured in the filtered seawater control, where cells did not exhibit fleeing behaviors. At initiation the model was seeded with a spatially uniform *H. akashiwo* abundance (100 cells ml^−1^), representing a below detection limit abundance. Ten replicate model iterations were run to incorporate variability in the movement parameters and averaged. Predicted abundance and distribution varied <1% among replicate model iterations. The model was evaluated using MatLAB (v. 7.10).

### Statistics

The Kolmogorov-Smirnov test was used to determine significant differences among distributions of swimming behaviors. A Repeated Measures Analysis of Variance (ANOVAR) was used to test for differences in *H. akashiwo* population distributions and swimming behaviors over time. A one-way ANOVA was used to compare growth rates between treatments or salinity structure. Vertical flux was calculated from time- and horizon-specific vertical velocity and cell density measurements. For all analyses the significance level was set to p<0.05.

## Supporting Information

Figure S1 **Diagram of experimental design and initial population distributions of predator and prey in the four treatments.** Salinity (psu) is indicated along the height of the tank. The first three treatments included a halocline (8–10 and 27–30 psu) and the last a linear gradient (27–30 psu). From left to right: (1) halocline with grazing *Favella* sp. (red triangle); (2) halocline with non-grazing *Favella* sp. (orange triangle), (3) halocline amended with filtrate (light red fill) from a grazing *Favella* sp. culture added to the bottom half of the tank; and (4) linear salinity gradient with grazing *Favella* sp. strain. Each treatment was observed in triplicate, independent tanks, along with triplicate *H. akashiwo* and *Favella* sp. only controls (not shown). Species distributions reflected their halo-tolerance: *Favella* sp. is stenohaline and can only persist at salinities >15 psu, in the bottom half of the halocline tank but distributed throughout the water column in the linear salinity gradient, while *H. akashiwo* (green circle) cells distributed throughout the experimental tank, irrespective of the salinity structure but aggregated to the halocline if present.
(TIF)
