# Supplementary figures and images for: Predator-Induced Fleeing Behaviors in Phytoplankton: A New Mechanism for Harmful Algal Bloom Formation?
Source: PLoS One. 2012 Sep 28;7(9):e46438. doi: 10.1371/journal.pone.0046438 (PMC3460921; doi:10.1371/journal.pone.0046438)

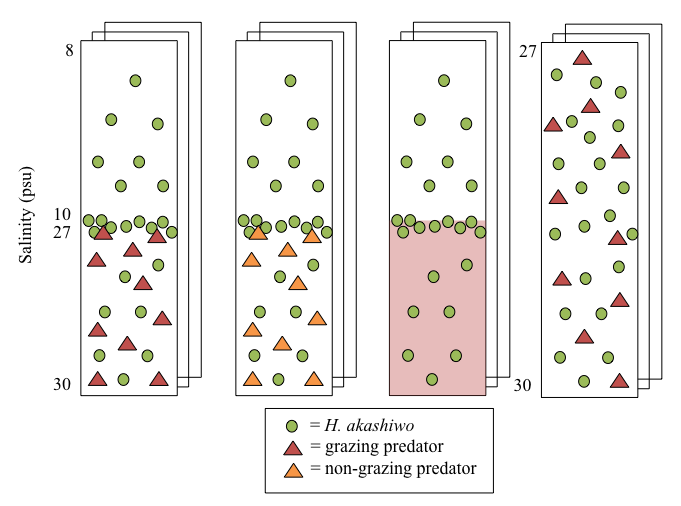

Supplement: Figure S1 — Diagram of experimental design and initial population distributions of predator and prey in the four treatments. Salinity (psu) is indicated along the height of the tank. The first three treatments included a halocline (8–10 and 27–30 psu) and the last a linear gradient (27–30 psu). From left to right: (1) halocline with grazing Favella sp. (red triangle); (2) halocline with non-grazing Favella sp. (orange triangle), (3) halocline amended with filtrate (light red fill) from a grazing Favella sp. culture added to the bottom half of the tank; and (4) linear salinity gradient with grazing Favella sp. strain. Each treatment was observed in triplicate, independent tanks, along with triplicate H. akashiwo and Favella sp. only controls (not shown). Species distributions reflected their halo-tolerance: Favella sp. is stenohaline and can only persist at salinities >15 psu, in the bottom half of the halocline tank but distributed throughout the water column in the linear salinity gradient, while H. akashiwo (green circle) cells distributed throughout the experimental tank, irrespective of the salinity structure but aggregated to the halocline if present. (TIF) [file pone.0046438.s001.tif]
